# Real-world validation of an AI-based non-mydriatic fundus camera in the diagnosis of diabetic retinopathy in Liberia

**DOI:** 10.3389/fdgth.2026.1938702

**Published:** 2026-09-24

**Authors:** Vickie Fayiah Sam, Mohammad Rizwan, John Chiemerie Douglas, Amrit Jha, Sirisha Senthil, Padmaja Kumari Rani

**Affiliations:** 1 LVPEI-Liberia Eye Center, John F. Kennedy Memorial Hospital, Monrovia, Liberia; 2 Brien Holden Institute of Optometry and Vision Sciences, L V Prasad Eye Institute, Hyderabad, India; 3 Kalathur Venugopal Reddy Glaucoma Institute, L V Prasad Eye Institute, Hyderabad, India; 4 Anant Bajaj Retina Institute, L V Prasad Eye Institute, Hyderabad, India

**Keywords:** artificial intelligence - AI, diabetic retinopathy (DR), fundus photography, Liberia (West Africa), low-resource setting, non-mydriatic imaging, retinal screening

## Abstract

**Introduction:**

Diabetic retinopathy (DR) is a leading cause of preventable vision loss, yet access to retinal screening remains limited in low-resource settings such as Liberia. This study evaluated the real-world diagnostic performance of an offline artificial intelligence (AI)-enabled smartphone-based non-mydriatic fundus camera for detecting referable diabetic retinopathy in a routine general outpatient screening setting in Liberia.

**Methods:**

This prospective study was conducted at the General Outpatient Department of John F. Kennedy Medical Center, Monrovia, Liberia. Adults aged 18 years and older were recruited through convenience sampling and underwent non-mydriatic fundus imaging using the Remidio Fundus on Phone (FOP-NM-10) integrated with an offline AI system. Fundus images were independently assessed by two trained optometrists using the International Clinical Diabetic Retinopathy (ICDR) classification, with disagreements adjudicated by a senior retinal ophthalmologist. Referable DR was defined as moderate non-proliferative DR, severe non-proliferative DR, or proliferative DR. Diagnostic performance was evaluated at the participant level among participants with at least one gradable eye.

**Results:**

A total of 1,612 participants were enrolled, of whom 1,357 were included in the final analytical cohort. Of these, 1,198 (88.3%) had at least one gradable eye and were included in the diagnostic-performance analysis. Eleven participants were classified as having referable DR by the human reference standard. The AI system correctly classified 10 of these 11 participants, yielding a sensitivity of 90.9% (95% CI, 58.7%–100%). Among 1,187 participants without referable DR, 1,183 were correctly classified as negative, resulting in a specificity of 99.7% (95% CI, 99.1%–99.9%). The positive predictive value was 71.4% (95% CI, 41.9%–91.6%), and the negative predictive value was 99.9% (95% CI, 99.5%–99.9%).

**Conclusions:**

In this prospective real-world study, the offline AI-based non-mydriatic fundus camera showed high sensitivity and specificity for detecting referable diabetic retinopathy among participants with at least one gradable eye in a general outpatient setting in Liberia. These findings support its feasibility for AI-assisted detection of referable DR in resource-limited settings.

## 1 Introduction

Liberia, a low-income country in West Africa with an estimated population of around 5.2 million, continues to face significant socio-economic and public health challenges (1, 2). Despite modest economic growth in recent years, the nation remains one of the least developed globally, with a gross domestic product (GDP) per capita among the lowest in the world and high levels of poverty that constrain access to essential services, including healthcare (3). The Liberian health system—still recovering from the effects of prolonged civil conflict and the devastating 2014–2016 Ebola epidemic—is characterized by limited infrastructure, shortages of trained healthcare providers, and inadequate diagnostic capacity, particularly in rural areas (4, 5).

Non-communicable diseases (NCDs) are an increasing burden in low-income countries such as Liberia, with diabetes prevalence increasing and posing additional strain on the fragile health system (6, 7). A population-based study using nationally representative data from five West African countries, including Liberia, reported an age- and sex-adjusted prevalence of type 2 diabetes mellitus of 6.2% in urban populations and 2.5% in rural populations (8).

A systematic review published in 2020 reported that the prevalence of undiagnosed diabetes in adults in the West African region is about 4.72% [95% confidence interval (CI), 2.64–6.80] (9). A substantial proportion of cases remain undiagnosed, highlighting gaps in early detection and chronic disease management. Among the complications of diabetes, diabetic retinopathy (DR) is a leading cause of blindness and requires timely detection to prevent irreversible damage (10). However, traditional retinal screening methods are often inaccessible or unaffordable for many Liberians, especially outside urban centers.

In West Africa, emerging evidence suggests that artificial intelligence (AI)-based systems can achieve strong diagnostic performance for detecting DR from fundus photographs. For example, a prospective study in Senegal using the Gaiha Prio Retino + ^TM^ AI software, reported sensitivities exceeding 92% and specificities of 99% when compared with ophthalmologist grading in hospital-based settings, conducted under controlled conditions, with limited data on real-world performance during routine outpatient screening, particularly in low-resource health systems (11). This study aims to validate the real-world diagnostic performance of an offline AI-based non-mydriatic fundus camera for detecting referable DR within a routine outpatient screening setting in Liberia.

## 2 Materials and methods

### 2.1 Study design and setting

This prospective study was conducted at the General Outpatient Department (OPD) of John F. Kennedy Medical Center (JFKMC), Monrovia, Liberia with referral linkage to the LVPEI-Liberia Eye Center. Study planning, protocol development, and preparation were initiated in April 2025, and the study protocol was submitted to the Institutional Review Board (IRB) of JFKMC. Ethical approval was granted on 15 June 2025 (Approval No. 202506/JFK00215), before initiation of the participant recruitment. Participant enrollment commenced on July 1st 2025 in accordance with the tenets of the Declaration of Helsinki. Written informed consent was obtained from all the participants before enrollment. The study evaluated the performance of an AI-based non-mydriatic fundus camera for detecting referable diabetic retinopathy under routine outpatient setting. This study is reported in accordance with the STARD-AI reporting guideline for diagnostic accuracy studies using artificial intelligence (12) (Supplementary Table 1:STARD-AI checklist).

As this study evaluates the real-world diagnostic accuracy of an already-marketed, regulatory-cleared AI system rather than a therapeutic or interventional trial, it was not prospectively registered in a clinical trial registry. The full study protocol is available from the corresponding author upon reasonable request.

### 2.2 Study population and sampling

Participants were recruited using convenience sampling from the General Outpatient Department of John F. Kennedy Medical Center during the study period. Screening was performed during the working hours when trained study personnel were available.

Sample size was estimated *a priori* (using the method of Buderer (13), which calculates the number of disease-positive subjects required to estimate sensitivity at a specified precision as:

$$n\;=\;Z^{2}\;\times \;Sn\left(1\;-\;Sn\right)/L^{2}$$

where Z is the standard normal deviate for the desired confidence level (1.96 for 95%), Sn/Sp is the anticipated sensitivity/specificity, and L is the desired absolute precision. Assuming a sensitivity of 90%, a specificity of 90%, a 95% confidence level, and an absolute precision of ±5 percentage points, this yielded a requirement of 139 disease-positive and 139 disease-negative participants. Diabetic retinopathy prevalence among the general population was assumed to be 10%, as no Liberia-specific published estimate was available at the time of protocol design; this would have required enrolling approximately 1,390 participants with diabetes to obtain sufficient disease-positive cases.

### 2.3 Inclusion and exclusion criteria

#### 2.3.1 Inclusion criteria

Participants aged 18 years or older who provided written informed consent were enrolled in the study.

#### 2.3.2 Exclusion criteria

Participants were excluded if they were unable to provide consent or were younger than 18 years, or had ocular conditions that prevented capture of sufficient quality fundus images. Exclusion was based on the inability to acquire interpretable fundus photographs rather than the presence of a specific ocular condition alone. These conditions included dense cataract causing media opacity, corneal opacity, severe pterygium obstructing visual axis, severe active ocular infection, recent ocular surgery, or other ocular conditions that prevented successful imaging.

Fundus imaging was attempted in participants with these conditions; however, sufficient quality fundus images could not be captured despite repeated attempts; these participants were excluded from the final analytical cohort. Participants with ocular pathologies were not excluded when sufficient quality fundus images could be captured and were therefore retained for analysis.

A total of 232 participants were excluded because fundus imaging could not be obtained: dense cataract (*n* = 198), severe pterygium obstructing the visual axis (*n* = 25), dense cataract with pterygium (*n* = 5), corneal opacity (*n* = 2), severe conjunctivitis (*n* = 1), and keratitis (*n* = 1).

### 2.4 Study procedures

#### 2.4.1 Ocular examination

All enrolled participants underwent a brief ocular assessment by optometrists with more than 5 years of experience as part of routine screening. The anterior segment was examined using torchlight evaluation, and intraocular pressure was measured using a handheld rebound tonometer (iCare IC100). Ocular findings were documented prior to fundus imaging.

### 2.5 Fundus imaging protocol

All fundus images were acquired under non-mydriatic conditions using the smartphone-based portable “Remidio Fundus on Phone non-mydriatic fundus camera (Remidio, Bengaluru, India), FOP NM 10” device integrated with an offline AI algorithm system. Disc and macula centered images with an approximately 40-degree field of view were captured for each eye through an undilated pupil. All images were processed automatically by Medios AI-DR integrated software in Remidio Fundus on Phone. During image acquisition, the integrated AI quality module automatically assessed image adequacy and provided an output of “sufficient” or “insufficient.” When an image was insufficient, the optometrist made up to two additional attempts to obtain a sufficient image quality. Participants in whom sufficient imaging quality could not be obtained because of predefined ocular conditions were excluded from the final analytic cohort. Participants with persistent insufficient imaging quality and/or ocular findings requiring further ophthalmic assessment were referred to the LVPEI-Liberia Eye Center for clinical evaluation, including dilated fundus examination where indicated. Ungradable images were not considered negative for referable DR.

### 2.6 Artificial intelligence system

Retinal images were acquired using the Remidio Fundus on Phone non-mydriatic fundus camera (FOP-NM 10; Remidio Innovative Solutions Pvt. Ltd., Bengaluru, India), with FOP software version 6.1.2, analysed offline using MediosHI 3-in-1 AI software version 3.0, released in November 2024. The AI algorithm and operating parameters were locked before study commencement, and no retraining, recalibration, or threshold optimization or other algorithm modification was performed using data from the present study. A prespecified operating threshold was used for disease classification and was not modified during the study.

The AI system first assessed image quality. Images were subsequently analysed for diabetic retinopathy (DR), glaucoma, and age-related macular degeneration (AMD). For DR, the AI generated positive or negative outputs corresponding to referable and non-referable DR, respectively. The present study evaluated only the DR output; glaucoma and AMD outputs were not included in the diagnostic-performance analysis. According to manufacturer-provided information, the underlying AI models were developed using a heterogeneous dataset of more than 200,000 retinal images, including established datasets such as EyePACS (14). Detailed demographic characteristics of development datasets, including the distribution of participants by age, sex, and ethnicity for the individual AI models, are proprietary and were not fully available to the investigators. No images or participants from the present Liberian study were used for algorithm development, training, fine-tuning, calibration, or threshold selection, and no known participant- or image-level overlap existed between the present study and the datasets used for algorithm training or previous validation.

Image acquisition typically required less than 1 min, and on-device AI processing required approximately 5 s per image. Study operators received structured training in device operation and image acquisition and demonstrated competency before independently acquiring study images. The FOP device and associated AI platform have regulatory authorisations in multiple jurisdictions, including EU MDR Class IIa certification for the Medios AI software, CDSCO regulation in India, FDA 510(k) clearance for the FOP device in the United States, and registration of the DR AI system in Singapore.

### 2.7 Reference standard and image grading

#### 2.7.1 Diabetic retinopathy classification

Diabetic retinopathy was graded from the fundus photographs according to the International Clinical Diabetic Retinopathy (ICDR) disease severity scale (15). Eyes were classified as having no DR, mild non-proliferative diabetic retinopathy (NPDR), moderate NPDR, severe NPDR, or proliferative diabetic retinopathy (PDR). For this study, referable DR was defined as moderate NPDR, severe NPDR, or PDR. Mild NPDR was classified as non-referable DR, while eyes without evidence of DR were classified as having no DR. Diabetic macular edema (DME) was assessed from the fundus photographs according to the clinical DME severity criteria described in the ICDR classification, based on the presence of retinal thickening and/or hard exudates involving the macular region. Because screening was performed using non-stereoscopic fundus photographs without OCT or stereoscopic imaging, DME assessment was limited to findings visible on the available fundus images.

#### 2.7.2 Human reference standard

The reference standard was established through a two-stage grading process performed by two trained optometrists with 5–10 years of clinical experience and formal training in diabetic retinopathy (DR) grading according to the International Clinical Diabetic Retinopathy (ICDR) guidelines. In cases of disagreement between the two primary graders on either image quality or DR grading, a senior retina ophthalmologist adjudicated the final decision, which was considered the reference standard for analysis. All fundus images were anonymized before grading, and graders were masked to AI results, participant clinical history, and each other’s assessments.

In the first stage, the two primary graders independently assessed fundus image quality using a predefined three-grade image quality classification system. Grade 1 images were considered gradable, while grade 2 and grade 3 images were classified as ungradable. Any disagreement in the image quality assessment was resolved through review by a senior retina ophthalmologist.

In the second stage, the graders evaluated the complete fundus image set for each participant, including images from both eyes when available, and independently assigned DR grades according to the ICDR disease severity scale. When the two optometrists disagreed, the case was reviewed by the senior retinal ophthalmologist, and the adjudicated decision was considered the final reference standard. The retina specialist reviewed only discrepant cases and additionally identified DR cases, and did not independently grade all participants. All adjudications were completed before statistical analysis.

For diagnostic-performance analysis, the final DR classification was converted to the participant level. The participant-level DR grade was based on the worse DR grade between the two eyes. If both eyes were gradable, the worse grade was assigned as the participant-level classification. If only one eye was gradable, classification was based on that eye. If both eyes were ungradable, the participant was classified as having ungradable images. For participants with only one available eye, classification was based on that eye when gradable; otherwise, the participant was classified as having ungradable images.

The AI index test and the human reference standard were derived from the same fundus photographs obtained at a single study visit; therefore, no time interval elapsed and no additional clinical intervention occurred between the index test and reference standard assessments.

#### 2.7.3 Image quality assessment

After the image acquisition, fundus images quality was independently assessed by trained human graders using a three-grade classification system. Grade 1 (gradable): Excellent quality with clear visualization of the optic disc and macula, visibility of third-order vessels, and only minor peripheral blur/glare/artifacts. Grade 2 (ungradable): Optic disc sufficiently visible but macula insufficiently visible; visibility of second-order or large vessels, mild blur, or artifacts affecting ≤1/3–1/2 of the image are allowed. Grade 3 (ungradable): Poor-quality images with insufficient visualization of the optic disc and macula; major blur or exposure prevented reliable grading. Only grade 1 (gradable) images were considered for inclusion in the diagnostic analysis, while grade 2 and 3 (ungradable) images were excluded from diagnostic performance analysis.

### 2.8 Statistical analysis

Statistical analysis was performed using IBM SPSS Statistics. The primary diagnostic performance analysis was conducted at the participant (patient) level and included participants with at least one gradable eye. Sensitivity, specificity, positive predictive value (PPV), and negative predictive value (NPV), with 95% confidence intervals, were calculated for referable DR.

Inter-grader agreement was assessed between two independent optometrist graders using Cohen’s kappa coefficient. Both graders independently evaluated 120 fundus images for image quality and 120 gradable fundus images for referable DR grading, with each grader masked to the other’s assessments. Agreement analysis was performed using the pre-adjudicated grading results. For image quality assessment, agreement was evaluated using the original three-level grading system (grade 1, gradable; grade 2, ungradable; grade 3, ungradable). For diabetic retinopathy grading, agreement was assessed using three categories: No DR, non-referable DR, and referable DR. Cohen’s kappa values with 95% confidence intervals were calculated.

## 3 Results

### 3.1 Study population

A total of 1,612 participants were initially enrolled. Figure 1 shows the study flow diagram. Twenty-three participants were excluded because they were younger than 18 years, and a further 232 participants were excluded because sufficient fundus image quality could not be obtained due to ocular conditions that prevented imaging. Thus, 1,357 (84%) participants were included in the final analysis, of whom 744 (54.8%) were female and 613 (45.2%) were male. The mean age was 44.15 ± 12.32 years (range: 18–89 years), with a 95% confidence interval (CI) of 43.5–44.8 years. The demographic, systemic, and ocular-gradability characteristics of the study population are summarized in Table 1.

**Figure 1:**
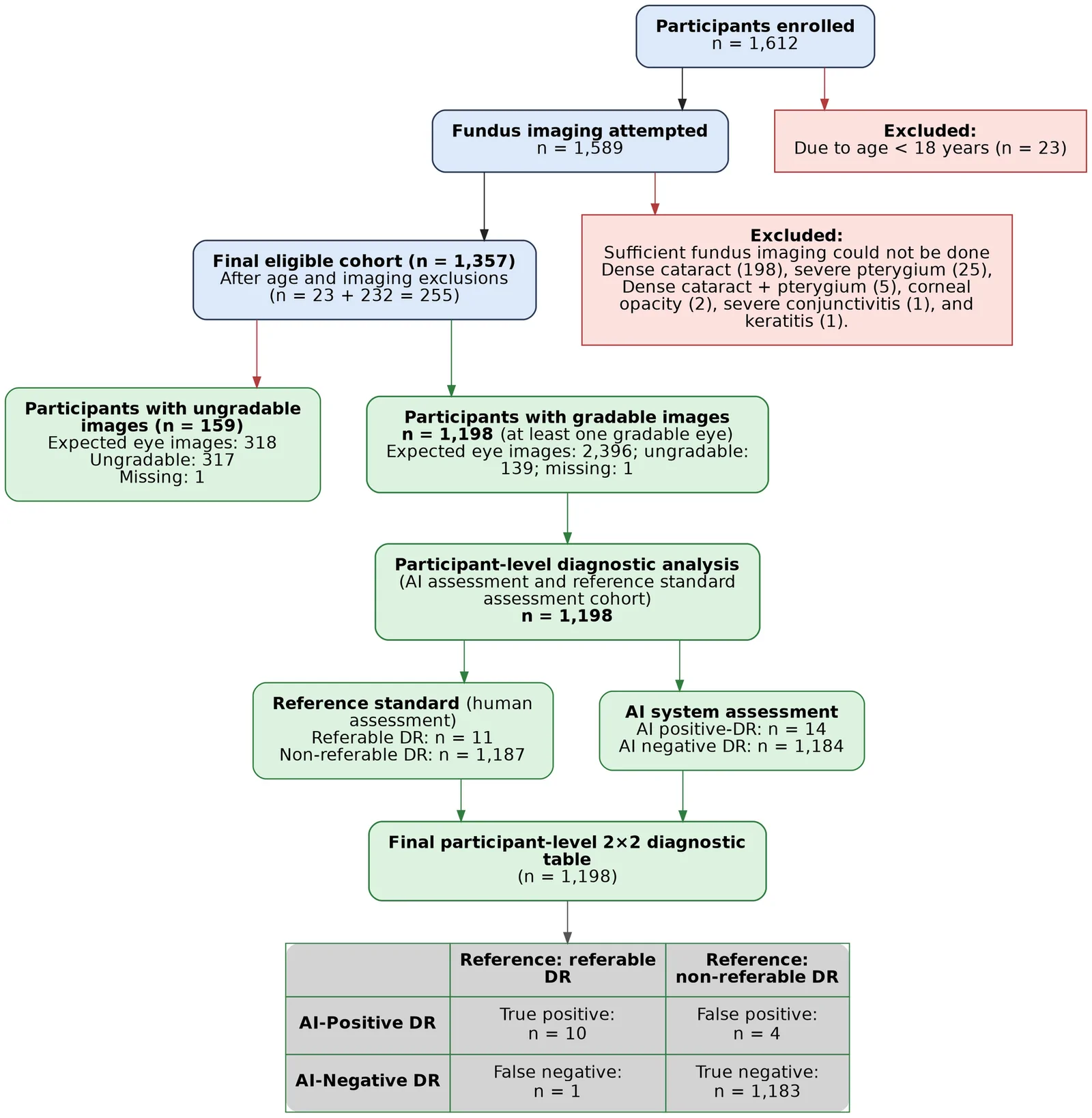
Study flow diagram.

**Table 1:** Demographic, systemic, and ocular characteristics of the study population.

Characteristic	Final eligible cohort (*n* = 1,357)	Known diabetes (*n* = 80)	Diagnostic-analysis cohort (*n* = 1,198)	No gradable eye (*n* = 159)
Age, years, mean ± SD	44.15 ± 12.32	58.90 ± 11.96	43.17 ± 11.77	51.48 ± 13.83
Age range, years	18–89	21–89	18–89	20–83
Sex, *n* (%)				
Female	744 (54.8)	44 (55.0)	670 (55.9)	74 (46.5)
Male	613 (45.2)	36 (45.0)	528 (44.1)	85 (53.5)
Systemic characteristics, *n* (%)				
Diabetes mellitus^a^	80 (5.9)	80 (100)	52 (4.3)	28 (17.6)
Hypertension^a^	193 (14.2)	39 (49)	152 (12.7)	41 (25.8)
Ocular characteristics, *n* (%)				
Cataract	80 (5.9)	9 (11)	71 (5.9)	9 (5.7)
Pterygium	93 (6.9)	8 (10)	85 (7.1)	8 (5.0)
Image gradability, *n* (%)				
≥1 gradable eye	1,198 (88.3)	52 (65.0)	1,198 (100)	0
No gradable eye	159 (11.7)	28 (35.0)	0	159 (100)
Referable DR, *n* (%)	—	8 (10.0)	11 (0.9)	—

Data are presented as *n* (%) unless otherwise indicated. The final eligible cohort comprised 1,357 participants. The diagnostic-analysis cohort included participants with at least one gradable eye (*n* = 1,198). Participants with no gradable eye (*n* = 159) were not included in the diagnostic-performance analysis. The known-diabetes group (*n* = 80) is a subgroup of the final eligible cohort and therefore overlaps with the diagnostic-analysis and no-gradable-eye groups. Three additional participants without previously known diabetes were subsequently confirmed to have diabetes during screening.

a Participants may have a history of more than one systemic disease.

At the time of screening, the majority of participants (1,123; 82.8%) reported no known systemic condition, whereas 234 (17.2%) reported at least one systemic condition. Among these, 41 (3.0%) reported diabetes mellitus (DM) only, 154 (11.3%) reported hypertension (HTN) only, and 39 (2.9%) reported both DM and HTN.

A total of 80 participants had self-reported history of DM. Referable DR was identified in 8 of these participants, corresponding to a prevalence of 10.0% (8/80). Among the 1,277 participants without self-reported history of DM, 3 (0.2%; 3/1,277) had retinal findings suggestive of DR during screening. These participants subsequently underwent fasting blood glucose testing, which confirmed diabetes mellitus in all three participants, with values of 222.1, 146.2, and 122.4 mg/dL, respectively (reference range: 70–115 mg/dL). Therefore, these participants were classified as newly confirmed cases of DM based on biochemical testing.

Overall, 83 participants had confirmed DM, comprising 80 participants with previously known DM and 3 newly confirmed cases.

### 3.2 Ophthalmic referral and dilated examination

Overall, 83 participants had confirmed DM, comprising 80 participants with previously known DM and 3 newly confirmed cases. When sufficient-quality images could not be captured after repeated attempts because of small pupil size or an uncooperative patient during fundus imaging, or because ocular findings required further evaluation, participants were referred to the LVPEI-Liberia Eye Center. Among 739 referred participants, 218 (29.5%) attended the eye clinic and completed a dilated fundus examination. Among these 218 participants who completed the dilated examination, the clinical findings included cataract (*n* = 88), pterygium (*n* = 39), glaucoma (*n* = 35), refractive error (*n* = 24), emmetropia (*n* = 31), age-related macular degeneration (AMD; *n* = 6), chorioretinitis (*n* = 4), conjunctivitis (*n* = 2), nerve palsy (*n* = 2), exotropia (*n* = 2), and proptosis (*n* = 1). Referable DR was identified in 10 participants. Because some participants had more than one ocular finding, the total number of clinical findings exceeded the number of participants examined.

Of the 10 participants diagnosed with referable DR during the dilated clinical examination, nine had already been identified as having referable DR by the human graders, whereas one additional participant was diagnosed with referable DR during the subsequent clinical examination despite having an ungradable fundus image grading and, therefore, was not classified as referable DR-positive by the human reference standard. Thus, the clinical examination identified one additional referable DR case that was not detectable from the fundus image.

### 3.3 Fundus image quality

A total of 3,164 fundus images were initially obtained from 1,589 participants. Of these, 2,318 (73.3%) images were gradable, 846 (26.7%) were ungradable, and 14 images were missing. These are image-level results. After exclusion of participants with predefined ocular conditions that prevented sufficient quality fundus imaging, 1,357 participants remained in the final analytical cohort. At the participant level 1,198 of 1,357 (88.3%) participants had at least one gradable eye, 2,256 of 2,396 images (94.2%) were gradable, 139 of 2,396 (5.8%) were ungradable and 1 image was missing. Among 159 of 1,357 (11.7%) participants with no gradable eye, 317 of 318 images (99.7%) were ungradable and 1 image was missing.

### 3.4 Ocular findings among eligible participants

Among the 1,357 participants included in the final analysis, 198 participants had at least one ocular finding identified during initial screening in the General OPD. The most frequent findings were pterygium (*n* = 98) and cataract (*n* = 85). Other ocular findings included squint (*n* = 6), conjunctivitis (*n* = 3), subconjunctival hemorrhage (SCH; *n* = 2), and ocular surface squamous neoplasia (OSSN; *n* = 2), ptosis (*n* = 1), and proptosis (*n* = 1). Participants with these ocular conditions remained eligible for study when sufficient quality images could be captured.

### 3.5 AI-based detection of referable DR

Among the 1,198 participants with at least one gradable eye included in the diagnostic-performance analysis, the AI system generated a positive output for referable DR in 14 participants. These AI-generated outputs were compared with the human reference standard to evaluate participant-level diagnostic performance. The AI platform also generated outputs for other ocular conditions, including glaucoma and AMD; however, these outputs were outside the scope of the present DR analysis.

### 3.6 Sensitivity and specificity of AI-based detection and human reference standard for referable diabetic retinopathy

Of the 1,357 participants included in the final analysis, 1,198 (88.3%) participants had at least one gradable eye and were included in the diagnostic performance analysis. Among these participants, 11 were classified as having referable DR according to the human reference standard. The AI system correctly classified 10 of these 11 participants, resulting in a sensitivity of 90.9% (10/11). Among the 1,187 participants without referable DR according to the reference standard, 1,183 were correctly classified as negative, yielding a specificity of 99.7% (1183/1187). The PPV was 71.4% (10/14), and the NPV was 99.9% (1183/1184). However, the relatively small number of referable DR-positive participants resulted in wide confidence intervals, particularly for sensitivity, indicating limited precision of the diagnostic-performance estimates. The participant-level diagnostic performance of an AI system for detecting referable DR is presented in Table 2.

**Table 2:** Participant-level diagnostic performance of the AI system for detection of referable diabetic retinopathy.

Variables	Human reference standard: Referable DR	Human reference standard: Non-referable DR	Total
AI positive DR	10 (TP)	4 (FP)	14
AI negative DR	1 (FN)	1,183 (TN)	1,184
Total	11	1,187	1,198
Diagnostic measure	Value (95% CI)		
Sensitivity	90.9% (58.7%–100%)		
Specificity	99.7% (99.1%–99.9%)		
PPV	71.4% (41.9%–91.6%)		
NPV	99.9% (99.5%–99.9%)		

Diagnostic performance was evaluated at the participant level among 1,198 participants with at least one gradable eye. Referable DR was defined as moderate NPDR, severe NPDR, or PDR according to the ICDR classification. For participants with two gradable eyes, the worse eye grade determined the participant-level classification; when only one eye was gradable, classification was based on that eye.

TP, true positive; FP, false positive; TN, true negative; FN, false negative; PPV, positive predictive value; NPV, negative predictive value.

### 3.7 Inter-grader agreement assessment

A total of 120 images were independently assessed by both optometrist graders before adjudication. For image quality grading, the observed agreement was 88.3% (106/120 images), with almost perfect agreement between graders (Cohen’s *κ* = 0.81; 95% CI, 0.71–0.90) (Supplementary Table 2). A separate set of 120 gradable images was independently assessed by the same graders for referable diabetic retinopathy grading, before adjudication. The observed agreement was 98.3% (118/120 images), with almost perfect agreement (Cohen’s *κ* = 0.84; 95% CI, 0.64–0.99) (Supplementary Table 2).

No adverse events were associated with performing the index test (fundus photography) or the reference-standard grading procedure, both of which are non-invasive.

## 4 Discussion

In this prospective study, we evaluated the diagnostic performance of an offline AI-based non-mydriatic fundus camera for detecting referable DR in a routine outpatient setting in Liberia. The AI system demonstrated high sensitivity of 90.9% and specificity of 99.7% for detecting referable DR compared with human reference standard, with a high negative predictive value of 99.9% and moderate positive predictive value of 71.4% among participants with at least one gradable eye.

The diagnostic performance observed in this study is broadly consistent with prior evaluations of AI-based referable DR screening systems. The study by Jain A et al. (16), conducted in a community screening setting, reported a sensitivity and specificity of an offline AI system for detecting referable DR 100.0% (95% CI, 94.72%–100.0%) and 89.55% (95% CI, 87.76%–91.16%). Similarly, Natarajan et al. (17) evaluated an offline Medios AI system integrated with the Remidio Non-Mydriatic Fundus on Phone in a prospective community-based study in India and reported a sensitivity of 100.0% (95% CI, 78.2%–100.0%) and specificity of 88.4% (95% CI, 83.2%–92.5%) for referable DR among 213 participants with gradable images. In the prospective SMART study, Sosale et al. (18) evaluated the offline Medios AI system using non-mydriatic Remidio fundus photographs in 900 individuals with diabetes. They reported a sensitivity of 93.0% (95% CI, 91.3%–94.7%) and specificity of 92.5% (95% CI, 90.8%–94.2%) for referable DR. The study by Varun Gulshan et al. (19), conducted as an in-clinic retrospective study, reported that in the EyePACS-1 dataset, the sensitivity was 97.5% and the specificity was 93.4%. In the Messidor dataset, the sensitivity was 96.1% and the specificity was 93.9% for detecting referable DR. In a multiethnic study, Daniel Shu Wei Ting et al. (20) reported a sensitivity and specificity of 90.5% and 91.6%, respectively, for referable DR, and 100% and 91.1%, respectively, for vision-threatening DR using a conventional desktop fundus camera. Similarly, a prospective study from Senegal by Mbaye S et al. (11) evaluated an offline AI-based screening system in a melanoderm population and reported a sensitivity of 92.31% and a specificity of 99.0% for referable DR. For vision-threatening DR, the sensitivity and specificity were 92.16% and 99.01%, respectively. These findings, derived from a West African clinical setting, are particularly relevant to the present study conducted in Liberia and demonstrate that high diagnostic accuracy of AI systems can be achieved in similar resource-constrained environments. Compared with these results, our study demonstrated comparable sensitivity (90.9%) and specificity (99.7%), which may reflect real-world variability in image quality, patient characteristics, and screening conditions in a routine outpatient setting.

Beyond these comparator studies, our findings are also usefully considered alongside independent, real-world implementation evidence. In a prospective community screening programme in Dominica using the same offline Medios AI/Remidio platform, Kemp et al. (21) reported a sensitivity of 77.5%–80.4% and specificity of 91.5% for referable DR — notably lower than observed in our cohort — illustrating that AI diagnostic performance can vary substantially across real-world deployments even when the same underlying algorithm and camera platform are used; consistent with this, Duggal et al. (22) recently demonstrated measurable differences between AI-DR performance under validation vs. routine clinical deployment, reinforcing the need for local, setting-specific validation before implementation. Image gradability, an important and setting-dependent determinant of real-world diagnostic yield, has similarly varied widely across programmes, from 11.7% of participants in our cohort to 23.6% of images in a large multicenter Chinese screening study [Zhang et al. (23)]. Notably, several of these comparator studies — including the Dominica and Chinese programmes and the SMART study cited above — were themselves community- or field-based rather than conducted under strictly controlled conditions, indicating that high AI-DR performance has already been demonstrated outside controlled research settings. The principal novelty of the present study therefore lies less in the general feasibility of real-world offline AI-DR screening, which has already been established across multiple settings, and more in providing, to our knowledge, among the first prospective diagnostic-accuracy data for this technology from Liberia, a low-resource West African health system with distinct infrastructure, DR prevalence, and screening-programme maturity.

A key finding of this study is the high negative predictive value (99.9%) observed in participants with gradable cases. In low resource settings such as Liberia, this finding suggests a potential role for AI-assisted screening as a supportive approach for identifying individuals unlikely to have referable DR among those with interpretable images. However, the impact of AI implementation on referral pathways, specialist workload, and patient outcomes was not evaluated in this study and requires further investigation. This is particularly relevant given the low prevalence of referable DR observed in our cohort, a factor known to influence predictive values in screening populations. In contrast, the moderate positive predictive value of 71.4% indicates the presence of false positive classifications, consistent with prior reports, and underscores the need for confirmatory human grading before clinical decision-making.

Importantly, our study extends existing evidence by evaluating AI performance for detecting referable diabetic retinopathy among participants with gradable cases in a general outpatient setting in Liberia. Previous studies evaluating AI-based DR screening have been conducted across a range of settings, including controlled datasets, clinical environments, and community-based screening programmes. The present study contributes additional evidence by evaluating an offline AI system in a routine general outpatient setting in Liberia, representing a healthcare environment with limited ophthalmic resources and infrastructure. Furthermore, the detection of previously undiagnosed referable DR among individuals without known diabetes highlights the potential value of opportunistic screening in general outpatient populations.

This study has several strengths. It represents a real-world validation of an offline AI-based referable DR detection system in West Africa. The use of masked, independent human graders with high inter-rater agreement strengthens the reliability of the reference standard. Furthermore, the inclusion of a general outpatient population provides insight into AI performance under routine screening conditions and enhances the relevance of the findings for similar healthcare settings.

Several limitations should be considered. First, this study was conducted at a single center in a general outpatient department, where the prevalence of referable DR was low. Only 11 of 1,198 participants (0.92%) had referable DR by reference standard — far below the 10% prevalence assumed at planning. This shortfall arose because the study was actually conducted in a general outpatient setting rather than one specifically targeting individuals with diabetes: only 80 of 1,198 participants self-reported diabetes, well short of the 1,390 statistically required, even though DR prevalence within this subgroup (8/80, 10.0%) closely matched our original assumption. Achieving the prespecified ±5-percentage-point precision at the observed prevalence would have required approximately 15,140 participants; the precision actually achieved was only ±15–18 points, consistent with the wide reported 95% confidence interval (58.7%–100%) in Table 2. These estimates should be interpreted cautiously. Second, a proportion of captured images were ungradable, reflecting an important limitation of non-mydriatic fundus photography in routine screening settings. Among the 1,357 participants in the final cohort, 1,198 (88.3%) had at least one gradable eye, whereas 159 (11.7%) had no gradable eye and were classified as ungradable. These participants were not considered negative for referable DR but were excluded from diagnostic performance analysis because the retinal status could not be reliably assessed. Therefore, the reported sensitivity, specificity, PPV, and NPV represent the performance of the AI system among participants with at least one gradable eye and should not be interpreted as the performance of the complete screening pathway, which also includes management of ungradable cases. Third, the reference standard was based on expert grading of the same smartphone-based fundus photographs evaluated by the AI system. Although images were independently graded by trained optometrists with retinal specialist adjudication for disagreements, this represents an image-based reference standard rather than an independent clinical reference standard. Consequently, both AI and human grading were subject to similar limitations related to image quality and restricted field of view. The agreement between automated AI image-quality assessment and human grading was not evaluated in this study and requires further investigation. Additionally, the study was not powered to evaluate subgroup-specific diagnostic performance across relevant demographic and clinical characteristics, including age, sex, diabetes status, cataract status, and image gradability. Therefore, potential differences in AI performance across these subgroups could not be assessed. Future studies with larger and more diverse populations are needed to evaluate the generalizability and fairness of AI-based DR screening across different participant groups.

## 5 Conclusion

In this prospective real-world study, an offline AI-based non-mydriatic fundus camera demonstrated promising diagnostic performance for detecting referable diabetic retinopathy among participants with gradable cases in a general outpatient setting in Liberia. These findings support the feasibility of AI-assisted detection of referable DR in resource-limited settings. However, the results should be interpreted cautiously due to the limited number of referable DR-positive participants, the presence of ungradable cases, and the single-center design.

## Data Availability

The raw data supporting the conclusions of this article will be made available by the authors, without undue reservation.
