# Supplementary material for: Real-world validation of an AI-based non-mydriatic fundus camera in the diagnosis of diabetic retinopathy in Liberia
Source: Front Digit Health. 2026 Sep 24;8:1938702. doi: 10.3389/fdgth.2026.1938702 (PMC13646051; doi:10.3389/fdgth.2026.1938702)
Supplement: Supplementary file 1 [file Table1.docx]

**STARD-AI Checklist**

*Standards for Reporting Diagnostic Accuracy Studies using Artificial Intelligence*

Manuscript title: Real-World Validation of an AI-Based Non-Mydriatic Fundus Camera in the Diagnosis of Diabetic Retinopathy in Liberia

Reference: Sounderajah V, Guni A, Liu X, Collins GS, Karthikesalingam A, Markar SR, Golub RM, Denniston AK, Shetty S, Moher D, Bossuyt PM, Darzi A, Ashrafian H; STARD-AI Steering Committee. The STARD-AI reporting guideline for diagnostic accuracy studies using artificial intelligence. Nat Med. 2025 Oct;31(10):3283–3289. doi:10.1038/s41591-025-03953-8

| **Section and Topic** | **Item No.** | **STARD-AI Item** | **Reported on page No. (this manuscript)** |
| --- | --- | --- | --- |
| **Title or abstract** | 1† | Identification as a study reporting AI-centered diagnostic accuracy and reporting at least one measure of accuracy within title or abstract | Page 1 (title); Page 2 (Abstract – Results: sensitivity, specificity, PPV, NPV reported) |
| **Abstract** | 2 | Structured summary of study design, methods, results and conclusions | Page 2 (Abstract, structured as Introduction/Methods/Results/Conclusions) |
| **Introduction** | 3† | Scientific and clinical background, including the intended use of the index test, whether it is novel or an established index test and its integration into an existing or new workflow, if applicable | Page 3 (Introduction, paragraphs 1–3, including prior AI-DR evidence from West Africa) |
|  | 4 | Study objectives and hypotheses | Page 3 (Introduction, final paragraph: study aim) |
| **Methods – Study design** | 5 | Whether data collection was planned before the index test and reference standard were performed (prospective study) or after (retrospective study) | Page 3 (Section 2.1: “This prospective study was conducted...”) |
| **Methods – Ethics** | 6* | Formal approval from an ethics committee. If not required, justify why. | Page 3 (Section 2.1: IRB approval, JFKMC, Approval No. 202506/JFK00215; Declaration of Helsinki; written informed consent) |
| **Methods – Participants** | 7† | Eligibility criteria: listing separate inclusion and exclusion criteria in the order that they are applied at both participant level and data level | Page 4 (Section 2.3: Inclusion and exclusion criteria) |
|  | 8 | On what basis potentially eligible participants were identified (such as symptoms, results from previous tests and inclusion in registry) | Pages 3–4 (Sections 2.1–2.2: attendees of the General OPD) |
|  | 9 | Where and when potentially eligible participants were identified (setting, location and dates) | Pages 3–4 (Section 2.1: JFKMC General OPD, Monrovia, Liberia; enrollment from 1 July 2025) |
|  | 10 | Whether participants formed a consecutive, random or convenience series | Page 4 (Section 2.2: convenience sampling) |
| **Methods – Dataset** | 11* | Source of the data and whether they have been routinely collected, specifically collected for the purpose of the study or acquired from an open-source repository | Pages 3–4 (study data prospectively/specifically collected); Page 5 (Section 2.6: AI development dataset, including EyePACS, an open-source repository) |
|  | 12* | Who undertook the annotations for the dataset (including experience levels and background) and how (within the same clinical context or in a post hoc fashion), if applicable | Page 6 (Section 2.7.2: two optometrists with 5–10 years' experience; adjudication by senior retinal ophthalmologist) |
|  | 13* | Devices (manufacturer and model) that were used to capture data; software (with version number) used to engineer the index test, highlighting the intended use | Page 5 (Sections 2.5–2.6: Remidio Fundus on Phone FOP-NM-10; FOP software v6.1.2; MediosHI 3-in-1 AI software v3.0) |
|  | 14* | Data acquisition protocols (for example, contrast protocol or reconstruction method for medical images) and details of data preprocessing, in sufficient detail to allow replication | Page 5 (Section 2.5: non-mydriatic, disc/macula-centered images, ~40° field of view, on-device AI quality assessment) |
| **Methods – Test methods** | 15a | Index test, in sufficient detail to allow replication | Pages 5–6 (Section 2.6: Artificial intelligence system) |
|  | 15b* | How the index test was developed, including any training, validation, testing and external evaluation, detailing sample sizes, when applicable | Pages 5–6 (Section 2.6: development dataset of >200,000 images including EyePACS; demographic composition of the development dataset stated as proprietary and not fully available) |
|  | 15c | Definition of and rationale for test positivity cutoffs or result categories of the index test, distinguishing prespecified from exploratory | Page 5 (Section 2.6: “A prespecified operating threshold was used...and was not modified during the study”) |
|  | 15d* | The specified end-user of the index test and the level of expertise required of users | Page 6 (Section 2.6: study operators/optometrists trained and assessed for competency before independent use) |
|  | 16a | Reference standard, in sufficient detail to allow replication | Pages 6–7 (Sections 2.7.1–2.7.2: ICDR grading by two optometrists with adjudication) |
|  | 16b | Rationale for choosing the reference standard (if alternatives exist) | Not explicitly stated as a rationale; ICDR classification is used as the accepted clinical grading standard (Page 6, Section 2.7.1) |
|  | 16c | Definition of and rationale for test positivity cutoffs or result categories of the reference standard, distinguishing prespecified from exploratory | Page 6 (Section 2.7.1: referable DR prespecified as moderate NPDR, severe NPDR or PDR) |
|  | 17a | Whether clinical information and reference standard results were available to the performers or readers of the index test | Page 6 (Section 2.7.2); index test (AI) is fully automated and processes only the fundus image, without access to clinical information |
|  | 17b | Whether clinical information and index test results were available to the assessors of the reference standard | Page 6 (Section 2.7.2: “graders were masked to AI results, participant clinical history, and each other's assessments”) |
| **Methods – Analysis** | 18 | Methods for estimating or comparing measures of diagnostic accuracy | Page 7 (Section 2.8: Statistical analysis) |
|  | 19 | How indeterminate index test or reference standard results were handled | Page 5 (ungradable images not considered negative for referable DR); Page 7 (excluded from diagnostic-performance analysis) |
|  | 20 | How missing data on the index test and reference standard were handled | Page 9 (Section 3.3: counts of missing/ungradable images reported and excluded from analysis) |
|  | 21 | Any analyses of variability in diagnostic accuracy, distinguishing prespecified from exploratory | Page 12 (Limitations: study was not powered for subgroup-specific analyses; none performed) |
|  | 22 | Intended sample size and how it was determined | Page 4 (a priori sample size calculation using the method of Buderer) |
|  | 23* | Details of any performance error analysis and algorithmic bias and fairness assessments, if undertaken | Page 12 (Limitations: subgroup fairness/generalizability assessment not undertaken; identified as a direction for future studies) |
| **Results – Participants and dataset** | 24 | Flow of participants, using a diagram | Page 8 (Section 3.1, referencing Figure 1); Page 16 (Figure 1 legend); Figure 1 |
|  | 25† | Baseline demographic, clinical and technical characteristics of training, validation and test sets, if applicable | Page 8 (Section 3.1) and Table 1 (Page 16); development-set characteristics not fully available (Page 6) |
|  | 26a | Distribution of severity of disease in those with the target condition | Table 2 (Page 17: 11 referable-DR participants); severity sub-distribution (moderate NPDR/severe NPDR/PDR) not separately reported |
|  | 26b | Distribution of alternative diagnoses in those without the target condition | Pages 8–9 (Sections 3.2 and 3.4: cataract, pterygium, glaucoma, refractive error and other ocular findings) |
|  | 27 | Time interval and any clinical interventions between index test and reference standard | Page 7 (Section 2.7.2: “The AI index test and the human reference standard were derived from the same fundus photographs obtained at a single study visit; therefore, no time interval elapsed and no additional clinical intervention occurred...”) |
|  | 28* | Whether the datasets represent the distribution of the target condition that one would expect from the intended use population | Pages 11–12 (Discussion/Limitations: observed referable-DR prevalence of 0.92%, far below the 10% assumed at planning) |
|  | 29* | For external evaluation on an independent dataset, an assessment of how this differs from the training, validation and test sets | Page 6 (Section 2.6: no image- or participant-level overlap between this study and the AI training/validation datasets) |
| **Results – Test results** | 30 | Cross-tabulation of the index test results (or their distribution) by the results of the reference standard | Table 2 (Page 17: 2×2 cross-tabulation, TP/FP/FN/TN) |
|  | 31 | Estimates of diagnostic accuracy and their precision (such as 95% confidence intervals) | Pages 9–10 (Section 3.6) and Table 2 (Page 17: sensitivity, specificity, PPV, NPV with 95% CI) |
|  | 32 | Any adverse events from performing the index test or the reference standard | Page 10 (Section 3.7: “No adverse events were associated with performing the index test (fundus photography) or the reference-standard grading procedure, both of which are non-invasive.”) |
| **Discussion** | 33 | Study limitations, including sources of potential bias, statistical uncertainty and generalizability | Pages 11–12 (Discussion: Limitations paragraph) |
|  | 34 | Implications for practice, including the intended use and clinical role of the index test | Page 11 (key finding/NPV discussion); Page 12 (Conclusion) |
|  | 35* | Ethical considerations and adherence to ethical standards associated with the use of the index test and issues of fairness | Page 3 (ethics approval and informed consent, Section 2.1); Page 12 (Limitations: fairness/generalizability across participant subgroups not assessed) |
| **Other information** | 36 | Registration number and name of registry | Page 4 (Section 2.1: not prospectively registered, as this is a diagnostic accuracy evaluation of an already-marketed, regulatory-cleared AI system rather than an interventional trial) |
|  | 37 | Where the full study protocol can be accessed | Page 4 (Section 2.1: “The full study protocol is available from the corresponding author upon reasonable request.”) |
|  | 38 | Sources of funding and other support; role of funders | Page 13 (Section 8, Funding: Hyderabad Eye Research Foundation) |
|  | 39* | Commercial interests, if applicable | Page 12 (Section 6, Conflict of interest: authors declare no conflict of interest) |
|  | 40a* | Availability of datasets and code, detailing any restrictions on their reuse and repurposing | Page 13 (Section 9, Data Availability Statement: AI algorithm/code proprietary and not publicly available; de-identified participant data available from corresponding author on reasonable request) |
|  | 40b* | Whether outputs are stored, auditable and available for evaluation, if necessary | Page 13 (Section 9, Data Availability Statement: individual-level AI outputs logged/retained and available for independent audit or evaluation on request) |

**New item introduced in STARD-AI (not present in STARD 2015). † Item modified from STARD 2015.*
