# Supplementary material for: Real-world validation of an AI-based non-mydriatic fundus camera in the diagnosis of diabetic retinopathy in Liberia
Source: Front Digit Health. 2026 Sep 24;8:1938702. doi: 10.3389/fdgth.2026.1938702 (PMC13646051; doi:10.3389/fdgth.2026.1938702)
Supplement: Supplementary file 2 [file Table2.docx]

**Supplementary Table 2**

| **Agreement matrix for fundus image quality grading between two independent graders (n = 120).** | | | | |
| --- | --- | --- | --- | --- |
|  | **Rater 2: Grade 1** | **Rater 2: Grade 2** | **Rater 2: Grade 3** | **Total** |
| Rater 1: Grade 1 | 60 | 2 | 0 | 62 |
| Rater 1: Grade 2 | 6 | 28 | 2 | 36 |
| Rater 1: Grade 3 | 0 | 4 | 18 | 22 |
| Total | 66 | 34 | 20 | 120 |

**Agreement matrix for referable diabetic retinopathy grading between two independent graders (n = 120).**

|  | **Rater 2: No DR (1)** | **Rater 2: Non-referable DR (2)** | **Rater 2: Referable DR (3)** | **Total** |
| --- | --- | --- | --- | --- |
| Rater 1: No DR (1) | 113 | 1 | 0 | 114 |
| Rater 1: Non-referable DR (2) | 0 | 1 | 1 | 2 |
| Rater 1: Referable DR (3) | 0 | 0 | 4 | 4 |
| Total | 113 | 2 | 5 | 120 |
